# Sestrin2 Regulates Osteoclastogenesis via the p62-TRAF6 Interaction

**DOI:** 10.3389/fcell.2021.646803

**Published:** 2021-03-26

**Authors:** Sue Young Oh, Namju Kang, Jung Yun Kang, Ki Woo Kim, Jong-Hoon Choi, Yu-Mi Yang, Dong Min Shin

**Affiliations:** ^1^Department of Oral Biology, Yonsei University College of Dentistry, Seoul, South Korea; ^2^BK21 FOUR Project, Yonsei University College of Dentistry, Seoul, South Korea; ^3^Department of Dental Hygiene, Yonsei University Wonju College of Medicine, Wonju, South Korea; ^4^Department of Orofacial Pain & Oral Medicine, Yonsei University College of Dentistry, Seoul, South Korea

**Keywords:** reactive oxygen species, autophagy inducer, osteoclast differentiation, antioxidant proteins, bone homeostasis

## Abstract

The receptor activator of nuclear factor-kappa B ligand (RANKL) mediates osteoclast differentiation and functions by inducing Ca^2+^ oscillations, activating mitogen-activated protein kinases (MAPKs), and activating nuclear factor of activated T-cells type c1 (NFATc1) via the RANK and tumor necrosis factor (TNF) receptor-associated factor 6 (TRAF6) interaction. Reactive oxygen species (ROS) also plays an important role during osteoclastogenesis and Sestrin2, an antioxidant, maintains cellular homeostasis upon stress injury via regulation of ROS, autophagy, and inflammation. However, the role of Sestrin2 in osteoclastogenesis remains unknown. In this study, we investigated the role of Sestrin2 in the RANKL-RANK-TRAF6 signaling pathway during osteoclast differentiation. Deletion of *Sestrin2* (*Sesn2*) increased bone mass and reduced the number of multinucleated osteoclasts on bone surfaces. RANKL-induced osteoclast differentiation and function decreased in *Sesn2* knockout (KO) bone marrow-derived monocytes/macrophages (BMMs) due to inhibition of NFATc1 expression, but osteoblastogenesis was not affected. mRNA expression of RANKL-induced specific osteoclastogenic genes and MAPK protein expression were lower in *Sesn2* KO BMMs than wild-type (WT) BMMs after RANKL treatment. However, the *Sesn2* deletion did not affect ROS generation or intracellular Ca^2+^ oscillations during osteoclastogenesis. In contrast, the interaction between TRAF6 and p62 was reduced during osteoclasts differentiation in *Sesn2* KO BMMs. The reduction in the TRAF6/p62 interaction and TRAP activity in osteoclastogenesis in *Sesn2* KO BMMs was recovered to the WT level upon expression of Flag-*Sesn2* in *Sesn2* KO BMMs. These results suggest that Sestrin2 has a novel role in bone homeostasis and osteoclasts differentiation through regulation of NFATc1 and the TRAF6/p62 interaction.

## Introduction

Bone is a living organ that renews throughout life via bone remodeling. Bone remodeling results from a balanced collaboration between bone formation by osteoblasts and bone resorption by osteoclasts. When the homeostatic equilibrium of this process is broken, bone diseases, such as periodontitis, osteoporosis, osteopetrosis, autoimmune arthritis, and bone tumors, develop (Takayanagi, 2007; Crockett et al., 2011; Okamoto et al., 2017; Tsukasaki and Takayanagi, 2019). Mature osteoclasts are differentiated multinucleated cells (MNCs) derived from hematopoietic cells of the mononuclear lineage; the two essential factors required for osteoclast formation are macrophage colony-stimulating factor (M-CSF) and receptor activator of nuclear factor-κB ligand (RANKL), which is secreted by osteoblasts (Boyle et al., 2003; Takayanagi, 2007). The interaction between RANKL and RANK receptor activates tumor necrosis factor (TNF) receptor-associated factor 6 (TRAF6). TRAF6 activates downstream signals, including nuclear factor-κB (NF-κB), and mitogen-activated protein kinases (MAPKs), such as extracellular regulated kinase (ERK), c-jun N-terminal kinase (JNK), and p38 (Takayanagi et al., 2002b). In addition, the RANKL-RANK interaction leads to an increase in the intracellular reactive oxygen species (ROS) level and to oscillations in the intracellular Ca^2+^ concentration ([Ca^2+^]_*i*_). Ultimately, RANKL-mediated ROS generation and [Ca^2+^]_*i*_ oscillations induce nuclear translocation of nuclear factor of activated T-cells cytoplasmic 1 (NFATc1), formation of MNCs, and bone resorption (Takayanagi et al., 2002a; Lee et al., 2005; Kim et al., 2010).

Ca^2+^ is an essential second messenger in universal signaling cascade and plays a critical role in cellular life and death decisions, such as cell proliferation, gene expression, contraction, secretion, metabolism, cell survival, and apoptosis (Hempel and Trebak, 2017). The Ca^2+^ signaling pathways associate with other cellular signaling systems, including the ROS pathway. Although ROS can damage cellular components, including protein, lipids, and DNA, at moderate levels, ROS function as crucial signaling molecules in some physiological processes via their oxidizing activity (Gorlach et al., 2015; Hempel and Trebak, 2017). ROS and Ca^2+^ signaling can be regarded as bidirectional actions because ROS can regulate cellular Ca^2+^ signaling and Ca^2+^ signaling is key for ROS production during bone remodeling (Agidigbi and Kim, 2019). Thus, dysfunction in one of these systems might affect the other system leading to harmful effects and various disorders (Lee et al., 2005; Kim et al., 2010, 2012, 2013; Srinivasan et al., 2010; Domazetovic et al., 2017).

Sestrins, a highly conserved protein family, are induced by various environmental stresses, including DNA damage, oxidative stress, and nutritional stress. Sestrins protect and maintain cell and tissues homeostasis upon stress injury by negatively regulating ROS accumulation and the mammalian target of rapamycin (mTOR) protein kinase signaling. There are three Sestrin isoforms in vertebrates: Sestrin1 (Sesn1, PA26), Sestrin2 (Sesn2, Hi95), and Sestrin3 (Sesn3). Sesn1 associates with autophagy-related genes and negatively regulates the mTOR complex 1 (mTORC1) and ROS production in immune cells. Sesn2, an antioxidant, activates AMP-activated protein kinase (AMPK) and inhibits mTORC1 signaling. Sesn3 also suppresses mTORC1 activity and maintains Akt activity by activating the AMPK-TSC1/2 signaling axis (Wang et al., 2019; Kim et al., 2020).

Human Sestrin2 (hSestrin2) consists of three subdomains: Sesn-A, Sesn-B, and Sesn-C. Sesn-A functions as an active alkyl hydroperoxide reductase and is essential for the antioxidant effect. Sesn-B contains a leucine-binding site and interacts with leucine. Sesn-C interacts with the Rag GTPase-activating protein complex to regulate AMPK and mTORC1 signaling (Kim H. et al., 2015; Saxton et al., 2016). Sesn2 also protects cells from oxidative stresses by activating autophagic regulators through the kelch-like ECH-associated protein 1 (Keap1)-nuclear factor erythroid-2-related factor 2 (Nrf2) pathway and through interaction with p62, an adaptor protein for autophagy (Bae et al., 2013; Rhee and Bae, 2015). p62 activates mTORC1-dependent nutrient sensing, NF-κB-mediated inflammatory responses, and the Nrf2-activated antioxidant defense in hepatocarcinoma cells (Taniguchi et al., 2016). Interestingly, several studies have shown that p62 is an atypical protein kinase C (aPKC) interacting protein that mediates the TRAF6-p62-aPKC signaling axis in RANKL-induced osteoclastogenesis (Duran et al., 2004; McManus and Roux, 2012; Li et al., 2014). Expression of NF-κB and MAPKs associate with activation of RANKL-RANK-TRAF6 downstream signaling, which regulates expression of NFATc1 and the functions of MNCs during osteoclastogenesis (Takayanagi et al., 2000; Boyle et al., 2003). Although it has been shown that Sesn2 protects cells from harmful stresses by inhibiting mTORC1 and ROS and by regulating autophagy via the Sesn2 and Keap1-p62 interaction, the regulatory role of Sesn2 during the maintenance of bone homeostasis remains unknown.

In this study, we used *Sesn2* knockout (KO) mice to investigate the role of Sesn2 in the RANK-TRAF6 downstream signaling pathway and during RANKL-induced osteoclasts differentiation and bone metabolism.

## Materials and Methods

### Reagents

Sesn2 plasmid (pcDNA3.1 Flag-Sesn2; plasmid #61868) was obtained from Addgene (Cambridge, MA, United States). Lentiviral pLVX-EF1α-IRES-Puro (EIP) vector (#631988) was obtained from Takara Bio United States, Inc. (Mountain View, CA, United States). Anti- Sesn2 antibodies were obtained from ProteinTech (Rosemont, IL, United States). Anti-NFATc1 and anti-TRAF6 antibodies were obtained from Santa Cruz Biotechnology (Santa Cruz, CA, United States). Anti-phosphor (p)-p38, anti-p-ERK, anti-p-JNK, and anti-IκBα antibodies were obtained from Cell Signaling (Beverly, MA, United States). Anti-p62 antibodies were obtained from Abnova (Taipei City, Taiwan). Anti-tubulin antibodies were obtained from Developmental Studies Hybridoma Bank (Iowa city, IA, United States). Fura-2/AM was purchased from Invitrogen (Carlsbad, CA, United States). Pluronic F-127 was obtained from Molecular Probes (Eugene, OR, United States). RANKL was purchased from R&D Systems (Minneapolis, MN, United States). All other chemicals were purchased from Sigma-Aldrich (St. Louis, MO, United States).

### Animals and Cell Culture

*Sesn2* KO mice in the C57BL/6 background were generated as described (Woo et al., 2009) and all animal care and experimental procedures complied with institutional guidelines and were approved by the Institutional Animal Care and Use Committee (IACUC) in Yonsei University (IACUC approval no. 2020-0031). The femur and tibia were isolated from 4 to 6-week-old male mice as described previously (Son et al., 2019). All cells derived from bone marrow of femur and tibia were collected and cultured in α-minimum essential medium (α-MEM) (Gibco, Grand Island, NY, United States) medium containing 10% fetal bovine serum (FBS) (Gibco) and 30 ng/mL M-CSF. The following day, non-adherent cells in media were collected and seeded on adequate plates containing 30 ng/mL M-CSF (PeproTech, Rocky Hill, NJ, United States). After 3 days, non-adherent cells were washed out with fresh media, and adherent cells were used as bone marrow-derived monocytes/macrophages (BMMs). Primary osteoblast cell cultures were prepared as described previously (Son et al., 2018). In brief, the calvariae of 1∼3-day-old mice were digested with an enzyme solution containing 0.1% collagenase and 0.1% dispase. The isolated osteoblastic cells were cultured for 4 days in α-MEM supplemented with 10% FBS and 1% antibiotic-antimycotic solution in a humidified incubator containing 5% CO_2_. Cells were plated at a density of 3 × 10^4^ cells/well in 24-well plates. Osteoblastic differentiation of cells was induced by 50 μg/ml ascorbic acid and 10 mM β-glycerophosphate when cells reached confluence.

### Micro-CT Scanning and Bone Histomorphometry

The distal femoral bone structure was analyzed by Advanced Institutes of Convergence Technology (Genoss Co., Ltd., Suwon, South Korea) after removing soft tissues. Two-dimensional images were analyzed using CTAn software (v.1.16, Bruker, Billerica, MA, United States). The dissected femurs of 13∼15-week-old male mice were fixed in 4% paraformaldehyde overnight at 4°C, decalcified in 15% ethylenediaminetetraacetic acid and embedded in paraffin. Paraffin-embedded sections were cut and stained with tartrate-resistant acid phosphatase (TRAP)/alkaline phosphatase (ALP) stain kit (Wako, Osaka, Japan) according to the manufacturer’s protocols. Staining was visualized with an Olympus IX71/F22PH microscope (Tokyo, Japan) and measurements performed with Bio-Quant software (Bioquant Image Analysis Co., Nashville, TN, United States).

### Alkaline Phosphatase (ALP) Activity Assay and Alizarin Red S (ARS) Staining

After osteogenic induction, cells were rinsed two times with phosphate buffered saline, fixed in 4% paraformaldehyde for 10 min, and then rinsed with deionized water three times. ALP and alizarin red S (ARS) were added into cells for the staining during 30 min. Images of each sample were acquired using a CCD camera. To quantify matrix mineralization, cells were washed once with PBS solution and incubated in 100 mM cetylpyridinium chloride for ARS and alkaline phosphatase yellow for ALP. The concentration of ARS was measured using a microplate spectrophotometer at a 562 nm wavelength and ALP activity was measured at the wavelength of 405 nm.

### Tartrate-Resistant Acid Phosphatase (TRAP) Stain Assay

Bone marrow-derived monocytes/macrophages of WT and *Sesn2* KO mice were seeded in 48-well plates at a concentration of 3 × 10^4^ cells/well and cultured in α-MEM containing 10% FBS with 30 ng/ml M-CSF and 60 ng/ml RANKL. The culture medium was replaced every 2 days. After 6 days, a TRAP stain assay was performed to confirm the cell differentiation rate. TRAP^+^ cells were stained using a Leukocyte Acid Phosphate Assay Kit (Sigma-Aldrich) by following the manufacturer’s procedure and number of TRAP^+^ cells (containing ≥3 nuclei) were counted.

### Resorption Assay

Bone marrow-derived monocytes/macrophages were seeded on Osteo Assay surface (Corning, Corning, NY, United States) and differentiated into osteoclasts. After 6 days, cells were washed with sodium hypochlorite solution for 1 h at room temperature. Bone slices were imaged, and resorption pits were photographed and analyzed by using Bio-Quant software.

### Real-Time RT-PCR Analysis

Total RNA was isolated by using Trizol reagent (Thermo Fisher Scientific, Waltham, MA, United States) according to the manufacturer’s instruction. Total RNA was reverse transcribed to cDNA with the use of MultiScribe^TM^ Reverse Transcriptase (Applied Biosystems, Foster City, CA, United States). Quantitative PCR was performed to measure the relative mRNA levels using the StepOnePlus Real-Time PCR System (Applied Biosystems) with a 20 μl reaction mix containing cDNA, 0.2 μM primers, and 10 μl of SensiFAST^TM^ SYBR^®^ Hi-ROX Kit (BIOLINE, London, United Kingdom). The relative amount of mRNA normalized to 18S was calculated using the delta-delta method. The following primers were used: cathepsin K (CTSK) forward: 5′-GAAGAAGACTCACCAGAAG CAG-3′, reverse: 5′-YCCAGGTTATGGGCAGAGATT-3′; tartrate-resistant acid phosphatase 5 (ACP5, TRAP) forward: 5′-GCAACATCCCCT GGTATGTG-3′, reverse: 5′-GCAAACGGTAGTAAGGGCTG-3′; dendrocyte expressed seven transmembrane protein (DC-STAMP) forward: 5′-GGGGACTTATGTGTTTCCACG-3′, reverse: 5′-ACAAAGCAACAGACTCCCAAAT-3′; d2 isoform of the vacuolar ATPase v0 domain (Atp6v0d2) forward: 5′-CTGGTTCGAGGATGCAAAGC-3′, reverse: 5′-GTTGCCATA GTCCGTGGTCTG-3′; osteoclast-associated receptor (OSCAR) forward: 5′-CCTAGCCTCATACCCCCAG-3′, reverse: 5′-CGTTGATCCCAGGAGTCACAA-3′; αv integrin forward: 5′-CCGTGGACTTCTTCGAGCC-3′, reverse: 5′-CT GTTGAATCAAACTCAATGGGC-3′; β3 integrin forward: 5′-CCACACGAGGCGTGAACTC-3′, reverse: 5′-CTTCAGGTT ACATCGGGGTGA-3′; macrophage colony-stimulating factor 1 receptor (mCsf1r, M-CSFR) forward: 5′-GGACCTACC GTTGTACCGAG-3′, reverse: 5′-CAAGAGTGGGCCGGATC TTT-3′; Sestrin2 (Sesn2) forward: 5′-GCAGATCTATGATCG TAGCGGA-3′, reverse: 5′-GCTCTAGATCAGGTCATGTAGC-3′; NFATc1 forward: 5′-CAAGTCTCACCACAGGGCTCAC TA-3′, reverse: 5′-GCGTGAGAGGTTCATTCTCCAAGT-3′; 18S forward: 5′-ACCGCAGCTAGGAATGGA-3′, reverse: 5′-GCCTCAGTTCCGAAAACCA-3′.

### Measurement of [Ca^2+^]_*i*_

The cells were seeded on cover glass in a 35-mm dishes (5 × 10^4^ cells/coverslip) and stimulated with RANKL for the indicated times. The cells were loaded with 5 μM Fura-2/AM and 0.05% Pluronic F-127 for 30 min in physiological salt solution (140 mM NaCl, 5 mM KCl, 1 mM MgCl_2_, 1 mM CaCl_2_, 10 mM HEPES, 10 mM glucose, 310 mOsm, pH 7.4) at room temperature. Fura-2/AM fluorescence was measured using excitation wavelengths of 340 and 380 nm, and emitted fluorescence 510 nm (Ratio = F_340_/F_380_) was collected and monitored at 2 s intervals using a CCD camera (Universal Imaging Co., Downingtown, PA, United States). All data were analyzed using MetaFluor software (Molecular Devices, San Jose, CA, United States).

### Measurement of Intracellular ROS

The cells were seeded in 12-well plate with cover glass at a density of 1.5 × 10^5^ cells/well. ROS was detected using the fluorescent probe 5-(and-6)-carboxy-2′,7′ dichlorofluorescein diacetate (DCFDA). The cells were exposed to 60 ng/ml RANKL and 10 μM DCFDA for 30 min and examined with a laser-scanning confocal microscope (model LSM 510; Carl Zeiss) with a GFP filter set. The mean relative fluorescence intensity for each field was measured with a Zeiss vision system and averaged.

### Western Blot and Immunoprecipitation

Whole cell lysates were extracted in RIPA buffer (Tech & Innovation, Seoul, South Korea), and centrifuged at 12,000 × *g* for 10 min at 4°C. Supernatant was collected, and protein was measured using Pierce BCA Protein Assay Kit (Thermo Fisher Scientific, Waltham, MA, United States). Extracts were subjected to 8–10% SDS-PAGE and transferred to polyvinylidene difluoride membranes (Immobilon-P, Millipore Corp., Bedford, MA, United States). The membranes were blocked with 5% skimmed milk and probed with primary antibodies in 5% BSA in TBS-T overnight at 4°C. The primary antibodies were used as follows: NFATc1 (1:750), Sesn2 (1:1000), p-JNK (1:1000), p-ERK (1:1000), p-p38 (1:1000), IκBα (1:1000), TRAF6 (1:1000), p62 (1:4000), actin (1:1000), and tubulin (1:5000). Membranes were washed and exposed to horseradish peroxidase-conjugated secondary antibodies for 1 h. The antigen-antibody complexes were detected with an ECL^TM^ Prime Western Blotting Detection Reagent (Amersham Biosciences, Piscataway, NJ, United States). Quantification was performed by densitometry using ImageJ. For immunoprecipitation, 700–1000 μg of protein was incubated with 1 μg anti–rabbit TRAF6 antibody at 4°C overnight with rotation. Protein G-Sepharose was added and incubated with rotation for 2 h at 4°C. Beads were collected by centrifugation at 5000 rpm for 30 s, boiled in 2× SDS sample buffer at 95–100 centigrade for 10 min, loaded on an SDS-PAGE, and analyzed by immunoblotting for TRAF6 and p62.

### DNA Transfection

Approximately 1∼5 × 10^5^ BMMs were seeded on a 35-mm dish and incubated in antibiotic-free medium. After 24 h, DNA was mixed with 250 μl of Lipofectamine 2000 (Invitrogen) and 250 μl of Opti-MEM, incubated for 20 min at room temperature before adding the cell. The transfected cells were assayed at 24 h after post-transfection.

### Vector Construction and Transfection Using Lentiviral System

A 1.4 kb full-length human Flag-Sesn2 cDNA was subcloned into the pLVX-EIP vector (Takara) according to the manufacturer’s instructions. The recombinant reaction was generated pLVX-EIP-Flag-Sesn2, which is engineered to express Flag-Sesn2, and pLVX-EIP was a control. Lentivirus packaging was performed according to the manufacturer’s instruction by co-transfection of these vectors and packaging mixtures (Addgene) into 293T cells using Lipofectamine 2000 and incubated for 24 h. The viral supernatant was harvested after 48–72 h, and titers were determined. Cultured BMMs were infected with the pLVX-EIP-Flag-Sesn2 or control virus pLVX-EIP and incubated for 24 h before treatment with 50 ng/ml RANKL for the indicated times to induce osteoclastogenesis.

### Statistical Analysis

Data were presented as mean ± standard error of the mean (S.E.M.) from at least 3 independent experiments. Statistical significance was determined by using a Student’s *t*-test or ANOVA test. Statistical significance was set at *p* < 0.05 level.

## Results

### Deficiency of *Sesn2* Increases Bone Mass

**FIGURE 1:**
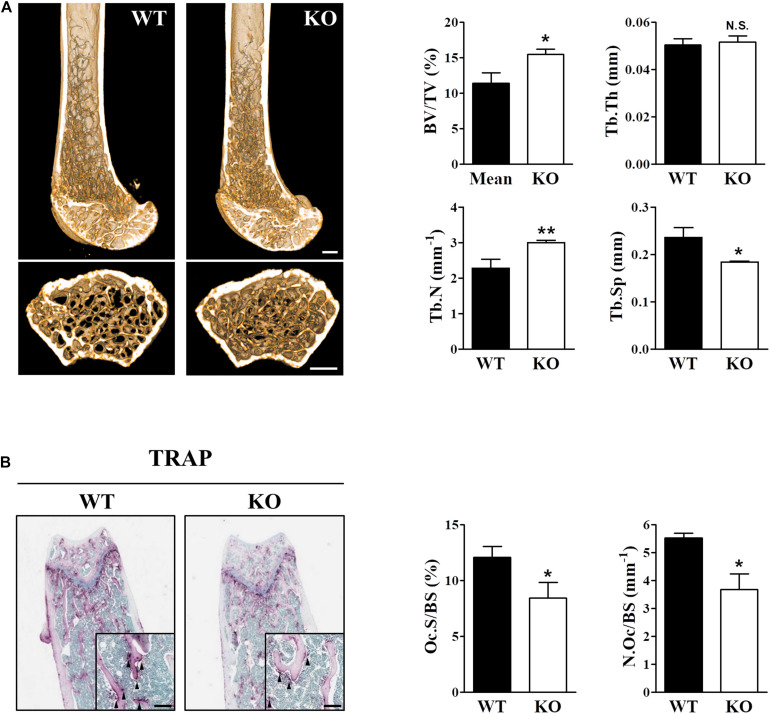
The skeletal phenotype is altered in *Sesn2* KO mice. For the *in vivo* experiments, distal femoral bones were isolated from WT and *Sesn2* KO male mice and analyzed histologically and by micro-computed tomography (μCT). **(A)** Micro-radiographic analyses of femur obtained from 13 to 15-week old WT (*n* = 6) and *Sesn2* KO mice (*n* = 9). The bone mass increased in *Sesn2* KO mice. Bone volume over total volume (BV/TV), trabecular thickness (Tb.Th), trabecular number (Tb.N), and trabecular separation (Tb.Sp) were expressed as mean ± S.E.M. *p < 0.05 and **p < 0.01 compared with WT mice. N.S. = not significant (Scale bar = 500 μm). **(B)** Trabecular surfaces of sectioned femurs from WT (*n* = 5) and *Sesn2* KO mice (*n* = 7) were TRAP-stained. TRAP^+^ osteoclasts along the trabecular surfaces on bone sections are indicated by arrows. There were fewer osteoclasts on the bone surface of *Sesn2* KO mice than WT mice. The percentages of TRAP-labeled osteoclast surface over bone surface (Oc.S/BS) were calculated, and the results are presented as fold-change relative to WT mice (Scale bar = 100 μm). Oc.S/BS and number of osteoclast over bone surface (N.Oc/BS) were measured using Bio-Quant software and expressed as mean ± S.E.M. *p < 0.05 compared with WT.

Intracellular ROS levels directly affected RANKL-induced osteoclast differentiation and bone density, and Sens2 functions as an antioxidant that suppresses ROS by activating AMPK and inhibiting mTORC1. Therefore, we examined whether the deletion of *Sesn2* alters bone phenotypes *in vivo* by dissecting the femurs from wild-type (WT) and *Sesn2* KO male mice, measuring bone mass by micro-radiographical analysis, and measuring osteoclastogenesis by histomorphological analysis using TRAP staining. The bone mass [bone volume/total volume (BV/TV)] and trabecular number (Tb.N) of *Sesn2* KO femurs were higher than WT femurs (∼35.6 and ∼31.8%, respectively), whereas trabecular separation (Tb.Sp) was ∼21.9% lower in femurs of *Sesn2* KO mice than WT femurs (Figure 1A). TRAP-stained bone sections were used to identify osteoclasts within femoral metaphysis and to quantify surface measurements. Osteoclast surface as a percentage of total bone surface (Oc.S/BS) and the number of osteoclasts over total bone surface (N.Oc/BS) in *Sesn2* KO sections were lower than WT sections (∼30.2 and ∼33.5%, respectively; Figure 1B). These results indicated that the increase in bone density in *Sens2* KO mice may result from decreases in the ratio and number for TRAP^+^ osteoclasts on the bone surface.

### Sens2 Modulates Osteoclast Differentiation and Function *in vitro*

**FIGURE 2:**
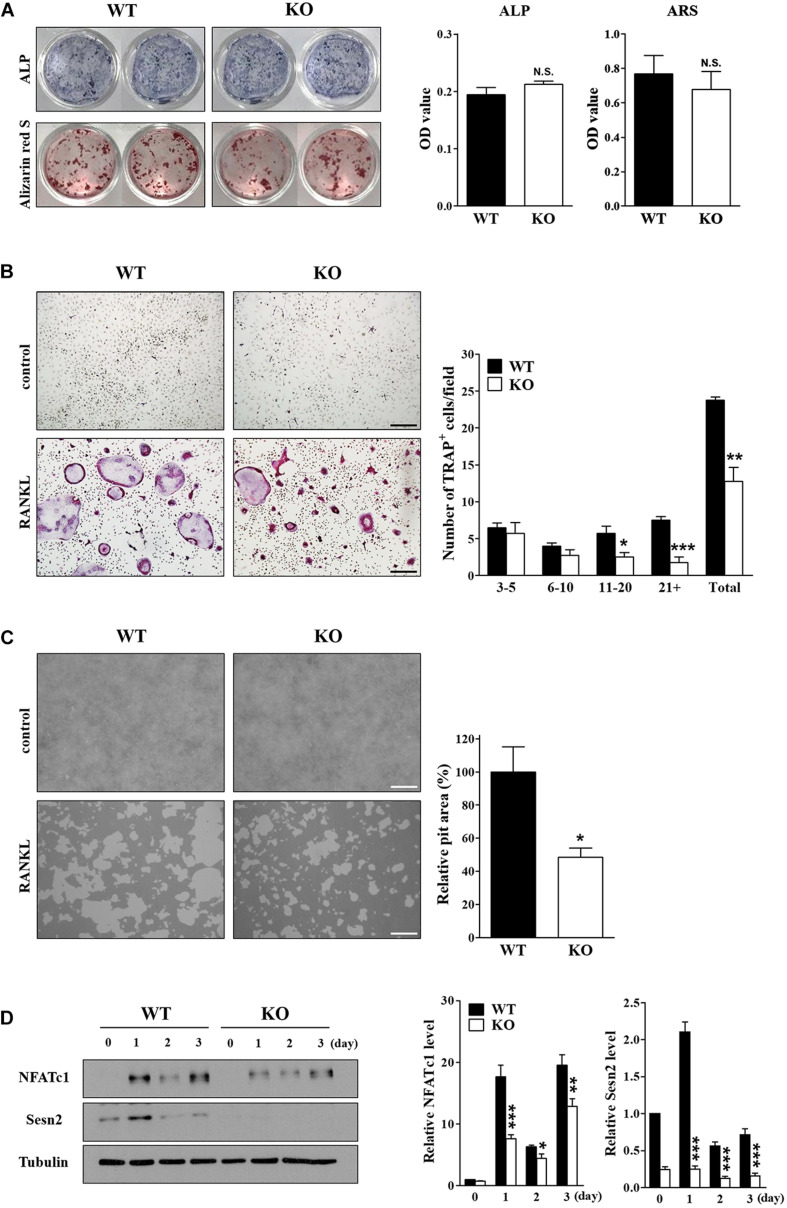
Deletion of *Sesn2* inhibits osteoclast differentiation. **(A)** Primary calvarial osteoblasts derived from WT and *Sesn2* KO mice were stained for ALP and ARS after 14 day of cell culture (*n* = 4). The *Sesn2* deletion had no effects on osteoblast differentiation in *Sesn2* KO mice. N.S. = not significant. **(B)** Primary BMMs derived from WT and *Sesn2* KO mice were TRAP-stained after 6 days of cell culture (*n* = 4). After staining, TRAP^+^ MNCs with more than three nuclei were scored as osteoclasts. Osteoclast differentiation was reduced in *Sesn2* KO mice (Scale bar = 50 μm). Data were expressed as mean ± S.E.M. *p < 0.05, **p < 0.01, and ***p < 0.001 compared with RANKL-treated WT mice. **(C)** BMMs were plated on a pit assay plate and cultured for 6 days (*n* = 4). The attached cells were removed and photographed under a light microscope. Pit areas were quantified using Bio-Quant software (Scale bar = 50 μm). RANKL-induced bone resorption was inhibited in *Sesn2* KO BMMs. The data were normalized to the resorptive area in RANKL-treated BMMs from WT mice. Results were expressed as mean ± S.E.M. *p < 0.05 compared with RANKL-treated WT mice. **(D)** Expression of NFATc1 (90∼110 kDa), Sestrin2 (54 kDa), and tubulin (50 kDa) in WT and *Sesn2* KO BMMs after RANKL stimulation were quantified by immunoblotting and ImageJ analysis (*n* = 6). Expression of NFATc1 was lower in *Sesn2* KO BMM cells than in WT BMM cells. Data were normalized to the expression in cells from WT mice and expressed as mean ± S.E.M. *p < 0.05, **p < 0.01, and ***p < 0.001 compared with WT.

We examined the effects of Sens2 on osteoblast differentiation in calvarial cells after treatments of ascorbic acid and β-glycerophosphate and on osteoclast differentiation of BMMs after RANKL treatment. ARS and ALP activity data showed that *Sesn2* expression did not affect osteoblastogenesis (Figure 2A). We then investigated the roles of Sens2 on osteoclastogenesis in M-SCF-treated and RANKL-treated WT and *Sesn2* KO BMMs. The number of TRAP^+^ osteoclasts, especially TRAP^+^ MNCs with more than 10 nuclei, was ∼46.3% lower in *Sesn2* KO BMMs than in WT BMMs (Figure 2B). To evaluate whether defects in osteoclastogenesis result in osteoclasts with impaired function in *Sesn2* KO mice, we performed a pit formation assay in WT and *Sesn2* KO BMMs. The formation of pits and resorbed areas were ∼51.5% lower in *Sesn2* KO BMMs than in WT BMMs (Figure 2C). To confirm the inhibitory effect of the *Sesn2* KO on osteoclastogenesis, we examined NFATc1 expression. Upon RANKL treatment, NFATc1 protein expression was induced and sustained in WT BMMs, whereas NFATc1 protein expression was reduced in *Sesn2* KO BMMs (∼88.0% in day 1 of RANKL treatments). The Sens2 protein level increased in WT BMMs on day 1 after RANKL treatment, but reduced thereafter (Figure 2D). These results demonstrated that Sens2 regulates osteoclasts, but not osteoblasts, differentiation and function.

### Deletion of *Sesn2* Correlates Negatively With Genes That Regulate Osteoclast Differentiation

**FIGURE 3:**
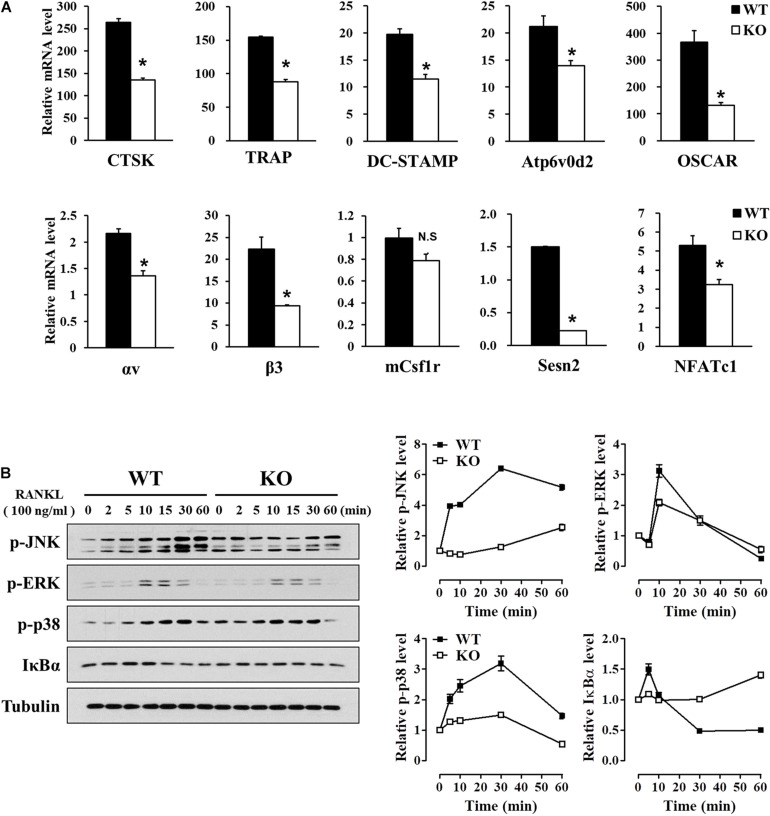
Reduced expression of osteoclast differentiation marker genes in *Sesn2* KO mice. **(A)** Expression of mRNAs for the master regulators of osteoclastogenesis were measured on 2 days after RANKL treatment. Osteoclastogenesis-related gene expression diminished in *Sesn2* KO BMMs during osteoclast differentiation (*n* = 4). Data were expressed as mean ± S.E.M. *p < 0.05 compared with WT. N.S. = not significant. **(B)** BMM cells were treated with 100 ng/ml RANKL for the period indicated. Protein expression of p-JNK (90∼110 kDa), p-ERK (44, 42 kDa), p-p38 (41 kDa), and IκBα (35 kDa) was quantified by immunoblotting and ImageJ analysis (*n* = 3). The RANKL-induced activation of the TRAF6 downstream pathways was reduced in *Sesn2* KO osteoclasts. Data were normalized to expression in WT cells and expressed as mean ± S.E.M.

Osteoclast differentiation, maturation, and function are regulated by specific osteoclastogenic genes, including NFATc1, CTSK, TRAP, DC-STAMP, Atp6v0d2, OSCAR, αvβ3 integrin, and mCsf1r (Boyle et al., 2003; Asagiri and Takayanagi, 2007; Okamoto et al., 2017; Tsukasaki and Takayanagi, 2019). We used real-time PCR to investigate the effects of *Sesn2* on expression of these genes during osteoclast differentiation of BMMs after 2 days of RANKL treatments. Except for mCsf1r, mRNA expression of most of the genes was lower in *Sesn2* KO BMMs indicating that Sens2 regulates maturation and function during osteoclast differentiation (Figure 3A). Expressions of NF-κB and MAPKs including ERK, JNK, and p38, correlate closely with activation of RANKL-TRAF6 downstream signals during osteoclastogenesis (Boyle et al., 2003). To confirm the effect of *Sesn2* on expressions of MAPKs, we examined protein expression of MAPKs and IκBα upon RANKL stimulation in WT and *Sesn2* KO BMMs. Activations of NF-κB by reduced degradation of IκBα proteins and MAPKs after RANKL treatment was lower in *Sesn2* KO BMMs than in WT BMMs. p-ERK expression increased in WT BMMs during the first 10 min of RANKL treatment, but expression diminished thereafter (Figure 3B). These results suggested that Sens2 acts on osteoclast differentiation and function by regulating osteoclastogenic genes in RANKL-TRAF6 downstream pathway.

### Sens2 Regulates Osteoclastogenesis Through Interaction Between TRAF6 and p62

**FIGURE 4:**
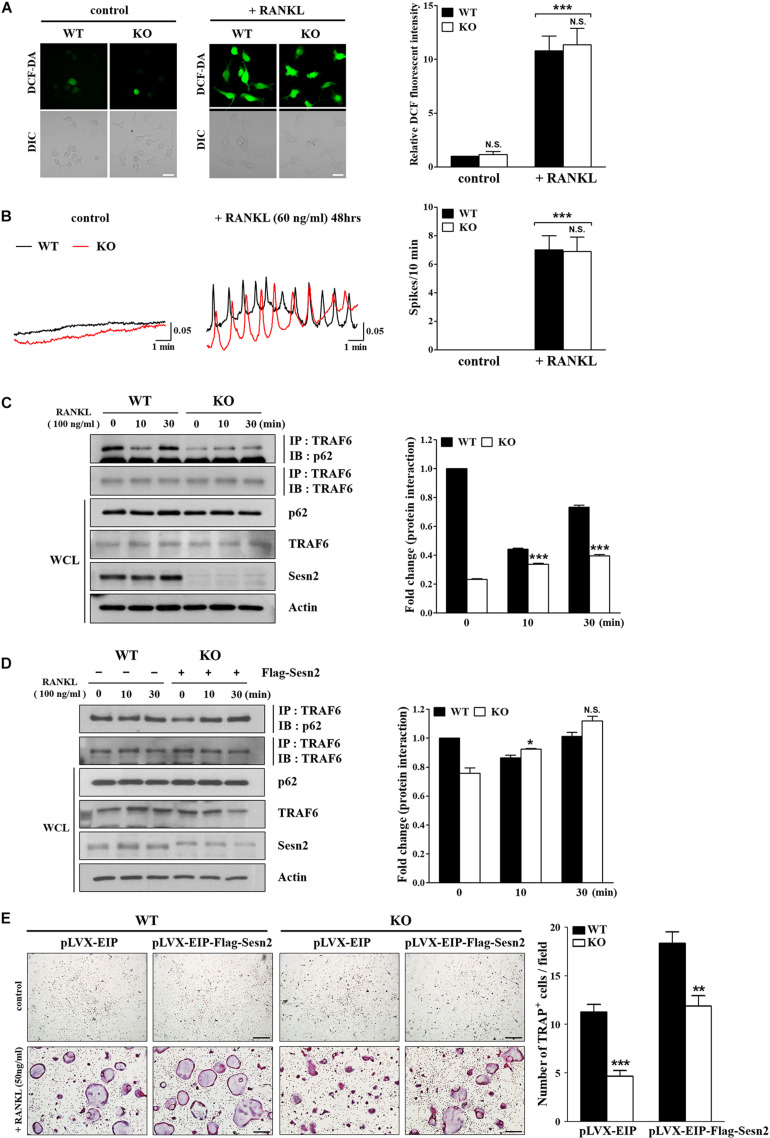
The p62 and TRAF6 interaction is inhibited in *Sesn2* KO mice. **(A)** RANKL-induced ROS generation was measured in WT and *Sesn2* KO BMMs (*n* = 7). Deletion of *Sesn2* had no effect on ROS generation in *Sesn2* KO BMMs. The data were normalized to the amount of ROS generated in WT cells and expressed as mean ± S.E.M. ***p < 0.001 compared with control. N.S. = not significant (Scale bar = 10 μm). **(B)** The number of RANKL-induced [Ca^2+^]_*i*_ oscillations were measured in WT and *Sesn2* KO BMMs (*n* = 5). Deletion of *Sesn2* had no effects on [Ca^2+^]_*i*_ oscillations. The data were expressed as means ± S.E.M. ***p < 0.001 compared with control. N.S. = not significant. **(C)** BMMs from WT and *Sesn2* KO mice were treated with RANKL for the period indicated, immunoprecipitated with anti-TRAF6 antibody, and the precipitates were immunoblotted for p62 (55 kDa) and TRAF6 (60 kDa; *n* = 3). The *Sesn2* deletion inhibited the interaction between p62 and TRAF6. Data were expressed as mean ± SEM. ***p < 0.001 compared with WT. **(D)** BMMs from *Sesn2* KO mice were transfected with Flag-*Sesn2* (*n* = 3). At 24 h post-transfection, cells were harvested and lysed, and cell lysates were subjected to immunoprecipitation with anti-TRAF6 antibody. The inhibition of interaction between p62 and TRAF6 in the *Sesn2* deletion was recovered by transfection of *Sesn2* KO BMMs with Flag-*Sesn2*. Data were expressed as mean ± SEM. *p < 0.05 compared with transfected WT. N.S. = not significant. **(E)** BMMs from WT and *Sesn2* KO mice were transfected with pLVX-EIP and pLVX-EIP-Flag-*Sesn2* (*n* = 3). At 24 h post-infection, cells were treated 50 ng/ml RANKL for 6 days, and then TRAP-stained. TRAP^+^ MNCs with more than ten nuclei were scored as osteoclasts. The reduction in TRAP^+^ osteoclasts in the *Sesn2* deletion was recovered to WT levels by transfection of pLVX-EIP-Flag-*Sesn2* into *Sesn2* KO BMMs when compared to transfection of pLVX-EIP into WT BMMs. Data were expressed as mean ± SEM (Scale bar = 50 μm). **p < 0.01 and ***p < 0.001 compared with RANKL-treated WT BMMs.

Sens2 suppresses the generation and action of ROS, thus we examined the effects of Sens2 on ROS generation and induction of [Ca^2+^]_*i*_ oscillations upon RANKL stimulations in WT and *Sesn2* KO BMMs. We observed that the lack of *Sesn2* did not affect the amount of ROS produced and the number of [Ca^2+^]_*i*_ oscillation spikes upon RANKL treatment (Figures 4A,B). Because Sesn2 activates autophagic degradation and interaction with p62 (Bae et al., 2013), we examined the interaction of TRAF6 with p62 in WT and *Sesn2* KO BMMs to determine if Sesn2 affects the TRAF6-p62 interaction and induction of osteoclastogenesis. Co-immunoprecipitation of p62 and TRAF6 upon RANKL stimulation showed that fewer TRAF6-p62 complexes were present in *Sesn2* KO BMMs than in WT BMMs, thus dissociation of TRAF6-p62 complexes were facilitated in *Sesn2* KO BMMs (Figure 4C). We also performed co-immunoprecipitation and TRAP assays after transient expression of *Sesn2* in WT and *Sesn2* KO BMM to determine whether TRAF6-p62 interactions increase upon recovery of Sesn2 expression and whether abnormal osteoclastogenesis is rescued by overexpression of *Sesn2*. Upon Sesn2 expression and RANKL stimulation, binding of TRAF6 to p62 in *Sesn2* KO BMMs increased to levels observed in WT BMMs (Figure 4D). In addition, upon *Sens2* overexpression, the abnormal osteoclast differentiation observed in *Sesn2* KO BMMs was returned to normal and levels of TRAP^+^ MNCs increased to levels found in WT BMMs (Figure 4E). These results indicated that Sesn2 regulates RANKL-induced osteoclast differentiation and function by mediating the interaction between TRAF6 and p62.

## Discussion

**FIGURE 5:**
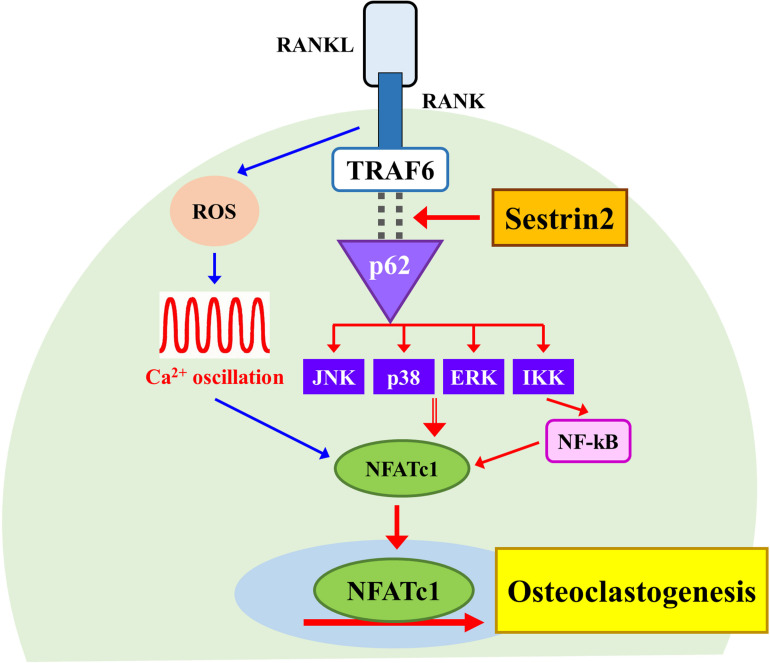
A schematic diagram for the regulatory mechanism of osteoclast differentiation by Sestrin2. The model shows the activation of NFATc1 requires Sestrin2-mediated activation for the TRAF6/p62 interaction (red arrows). The sequence of events activated by RANKL also induces the ROS generation and Ca^2+^ oscillations that are required for the activation of NFATc1 regardless of Sesstrin2 (blue arrows).

In this study, we demonstrated a novel role of Sesn2 to regulate interactions between TRAF6 and p62 during osteoclastogenesis. We also provided evidence that Sesn2 modulates the activities of NF-κB, MAPKs, as well as NFATc1 downstream signaling during osteoclasts differentiation. *Sesn2* KO mice had an high bone mass phenotype caused by a reduction in osteoclast differentiation through inhibition of expression of the osteoclastogenic genes NF-κB, MAPKs, and NFATc1, but not dependent on the rate of osteoblast differentiation or changes in intracellular ROS and Ca^2+^ levels (Figure 5). These findings suggest that Sesn2 regulates RANKL-induced NFATc1 activation during late stage osteoclast differentiation through the TRAF6/p62 interaction-mediated NF-κB and MAPKs pathways.

It has been shown that increases in [Ca^2+^]_*i*_ and induction of Ca^2+^ oscillations play important roles in RANKL-induced osteoclastogenesis (Takayanagi et al., 2002a). Increases in ROS through the RANKL-RANK-TRAF6 signaling axis activate MAPKs and differentiation to multinucleated osteoclasts, and these ROS-induced effects are inhibit by antioxidants, such as nicotinamide adenine dinucleotide phosphate (NADPH) oxidases 1 (NOX1) and Rac1, an activator of the various NOX family members (Lee et al., 2005). We demonstrated previously that Rac1-mediated ROS levels increased in a deletion mutant of peroxiredoxin II, a thiol-based peroxide reductase, and induced Ca^2+^ oscillations, as well as osteoclast differentiation and function during osteoclastogenesis (Kim et al., 2010). Previous reports on ROS production and RANKL-induced osteoclastogenesis have shown that ROS levels regulate expression and activity of differentiation factors during osteoclastogenesis, thereby determining the number of mature osteoclasts and bone density, but that ROS levels do not affect osteoblastogenesis (Kim et al., 2010, Kim J.H. et al., 2015; Goettsch et al., 2013; Hyeon et al., 2013; Kang and Kim, 2016; Lee et al., 2019; Ng et al., 2019; Han et al., 2020). Lee et al. (2019) has recently reported that reduction in osteoclast differentiation and increased bone density through regulation of ROS signaling result from reduced RANKL secretion by osteoblasts. However, we found that Sesn2 regulated osteoclastogenesis through the NF-κB and MAPK pathways and that Sesn2 did not affect osteoblast differentiation, ROS generation, or induction of Ca^2+^ oscillations. Activations of the antioxidant enzymes NAD-dependent deacetylase sirtuin 3 (Sirt3) and superoxide dismutase 2 (SOD2) inhibited osteoclastogenic genes and NFATc1 expression, formation of mature osteoclasts, and osteoclast functional activity by reducing mitochondrial ROS production (Kim et al., 2017). On the other hand, knock-down of Sesn2 increased lipopolysaccharide-induced ROS production and apoptosis by inhibiting SOD2 and catalase activity in heart (Hwang et al., 2018). Therefore, Sesn2 may exert its antioxidant effects via multiple regulatory pathways.

A previous report showed that the TRAF6/p62 interaction and activation of the NF-kB and/or MAPK pathways play important roles in RANKL-induced osteoclast differentiation (Duran et al., 2004). CYLD, a de-ubiquitinating enzyme, physically interacts with p62, the CYLD/TRAF6 complex negatively regulates TRAF6 ubiquitination and regulates the sustained inhibitory actions of NF-kB and NFATc1 during RANKL-induced osteoclastogenesis (Jin et al., 2008). Bae et al. (2013) demonstrated that Sesn2 interacts with Keap1, p62, and ubiquitin ligase and that the antioxidant activity of Sesn2 is mediated by activation of Nrf2 and p62-dependent autophagic degradation of Keap1 when ROS is increased as a result of an increase in mTORC1 activity and ER stress due to environmental stresses. According to these reports, it has a possibility to regulate the osteoclastogenesis via interactions between p62/Sesn2 and TRAF6/p62. Although we did not confirmed the Sesn2 interaction with TRAF6 or p62 under RANKL-induced osteoclast differentiation, our results combined with previous reports suggest that Sesn2 promotes a strong interaction between TRAF6 and p62 and promotes RANKL-induced osteoclastogenesis via activations of NF-κB and MAPK downstream signaling pathways. In addition, to protect cells against endotoxicity, such as inflammation and aging, Sesn2 is activated by an increase in oxidative stress-induced Nrf2 and AP-1 via activation of toll-like receptors, by inhibition of ubiquitin-mediated Sestrin degradation, and by an increase in Sestrin-MAPK complexes in immune cells (Wang et al., 2019; Kim et al., 2020). Further, it has been shown that p62 is highly expressed in osteoclasts in periapicitis and periodontitis models and that NOX4 is highly expressed in bones obtained from patients with osteoporosis and Paget disease, a focal disorder of enhanced bone remodeling (Goettsch et al., 2013; Li et al., 2014). Recently, it has been reported that Sestrin expression is suppressed in knee joint cartilage obtained from osteoarthritis and aging donors (Shen et al., 2017). Other reports are also showed that Sesn2 expression reduction in skeletal muscles of old mice is increased expression and induced autophagy by physical exercise, and Sesn1 and Sesn2 expression are increased by acute aerobic exercise in skeletal muscle of young mice (Lenhare et al., 2017; Crisol et al., 2018). Therefore, we suggest that Sesn2 protects against stimulation of the immune response and aging.

In summary, our results provide strong evidence for the role of Sesn2 in regulation of RANKL-induced osteoclasts differentiation and bone metabolism via the NF-κB and MAPK pathways and the TRAF6/p62 interactions. Specifically, our findings suggest that Sesn2 regulates bone remodeling by balancing autophagy and antioxidant signaling. We believe that our findings may be clinically relevance for pathological conditions of the immune and skeletal systems. We demonstrated the essential roles of Sesn2 in the TRAF6/p62 downstream signaling pathway during RANKL-induced osteoclast differentiation. These findings suggest a novel and potent therapeutic target for various bone and skeletal disorders caused by the abnormal formation of osteoclasts.

## Data Availability Statement

The raw data supporting the conclusions of this article will be made available by the authors, without undue reservation.

## Ethics Statement

The animal study was reviewed and approved by the Institutional Animal Care and Use Committee (IACUC) in Yonsei University (IACUC approval no. 2020-0031).

## Author Contributions

SYO and NK contributed to the data acquisition, analysis, and interpretation and drafted the manuscript. YMY contributed to the data acquisition, analysis, and interpretation, and drafted and critically revised the manuscript. JYK contributed to the conception and drafted the manuscript. KWK and JHC contributed to conception and revised the manuscript. DMS contributed to conception, design, and data interpretation and critically revised the manuscript. All authors gave final approval and agreed to be accountable for all aspects of the work.

## Conflict of Interest

The authors declare that the research was conducted in the absence of any commercial or financial relationships that could be constructed as a potential conflict of interest.
