# Supplementary material for: Sestrin2 Regulates Osteoclastogenesis via the p62-TRAF6 Interaction
Source: Front Cell Dev Biol. 2021 Mar 26;9:646803. doi: 10.3389/fcell.2021.646803 (PMC8033026; doi:10.3389/fcell.2021.646803)

**Original data for Western blots in Figure 2D**


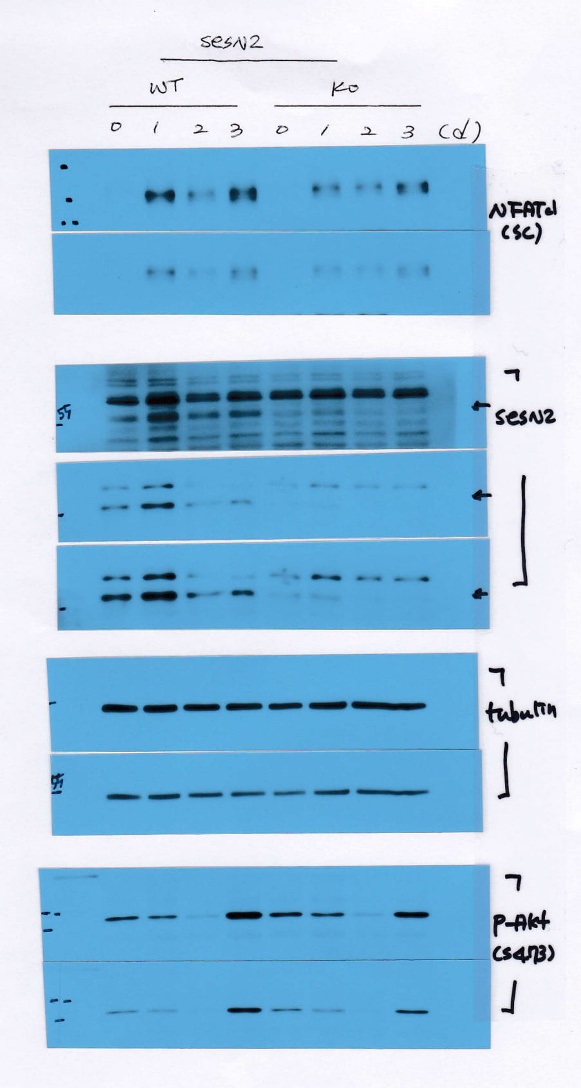


**Original data for Western blots in Figure 3B**


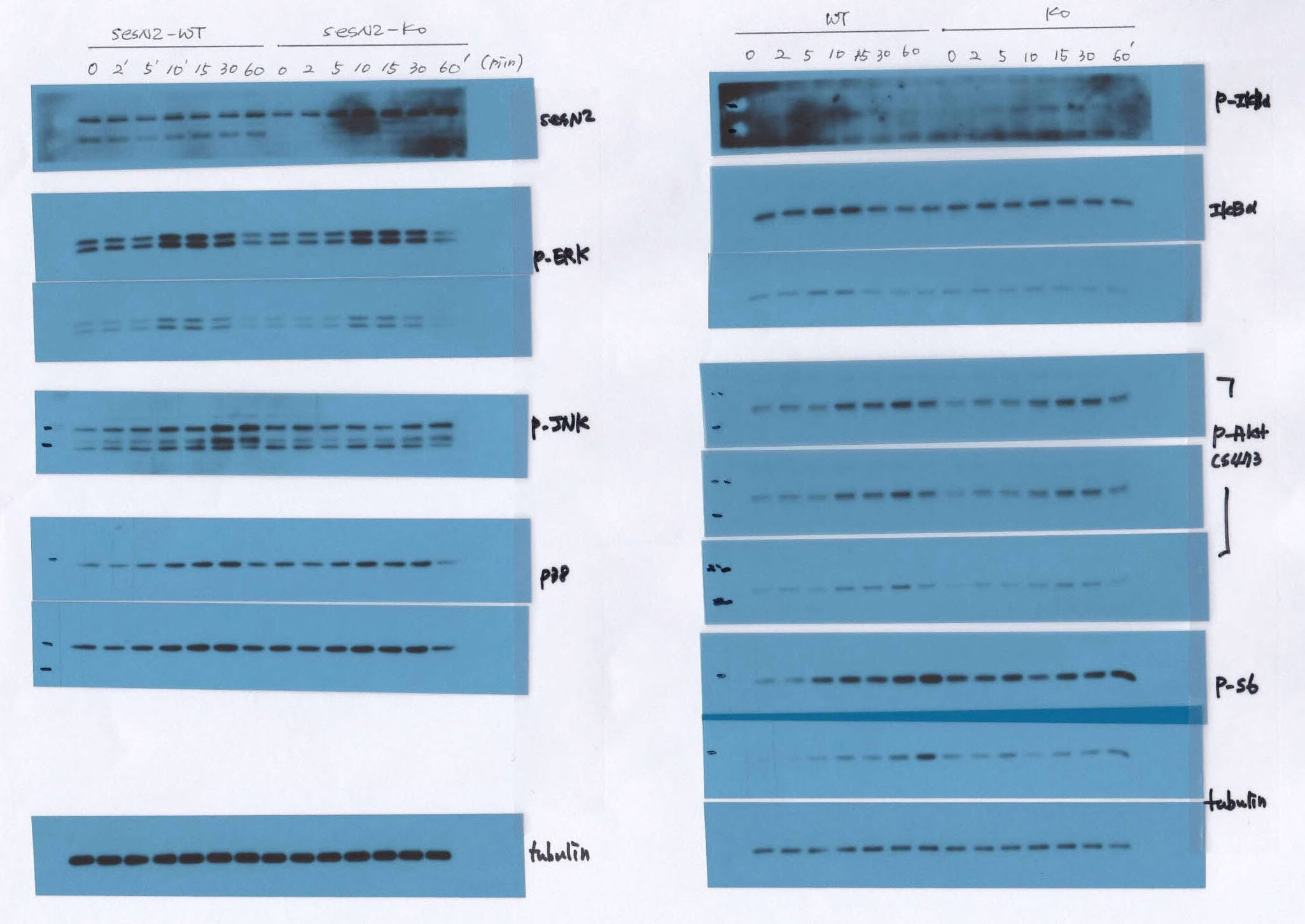


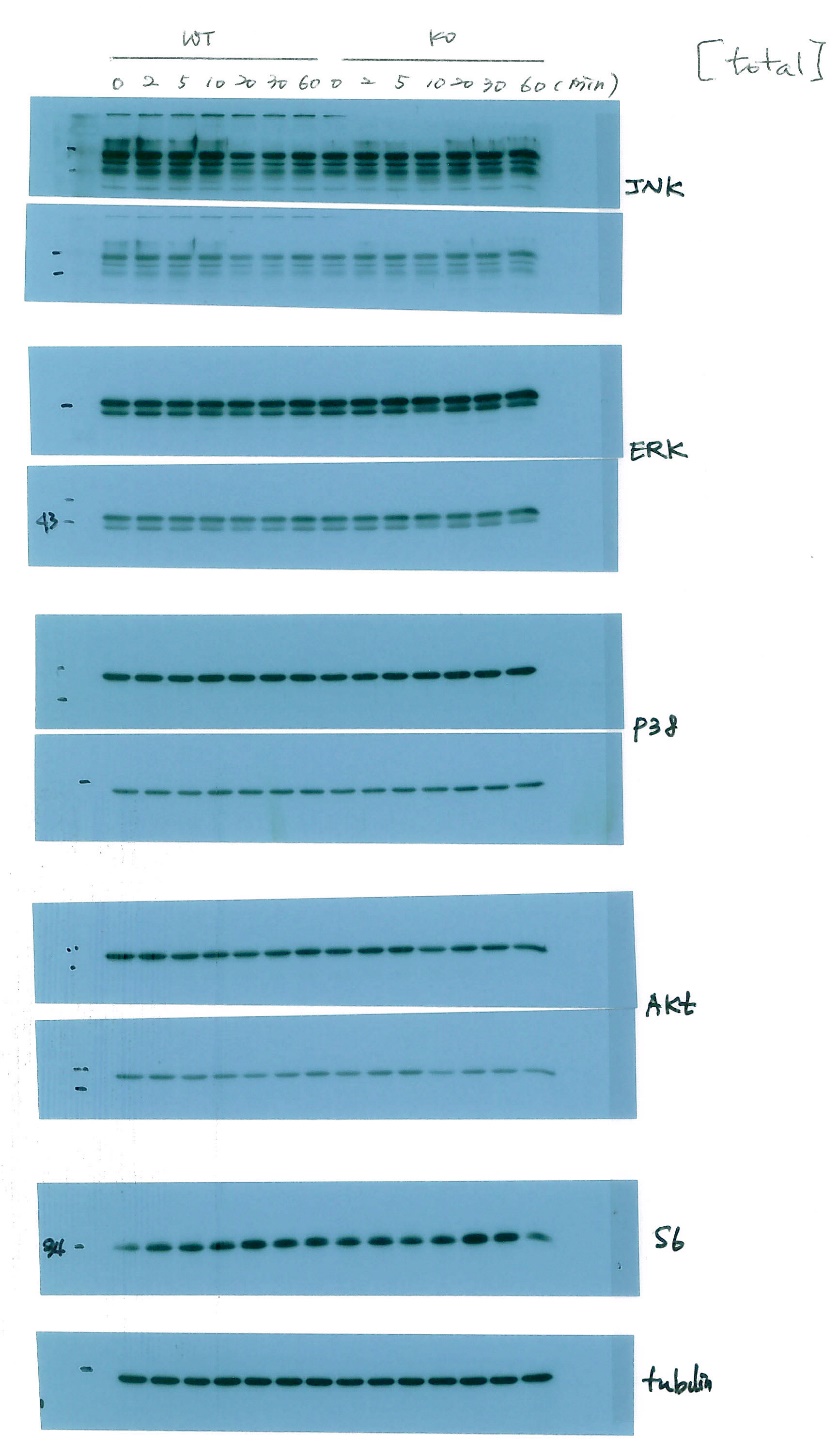


**Original data for Western blots in Figure 4C**


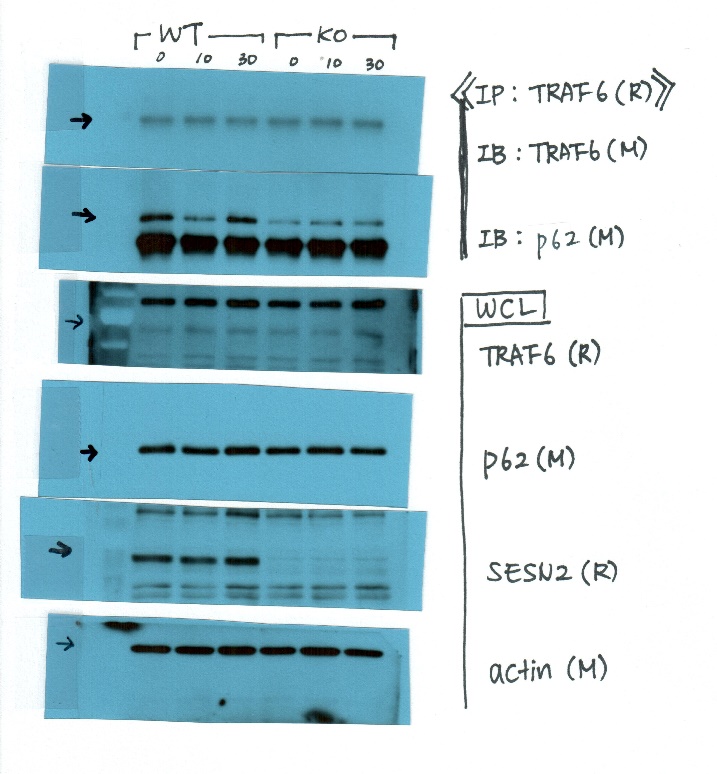


**Original data for Western blots in Figure 4D**


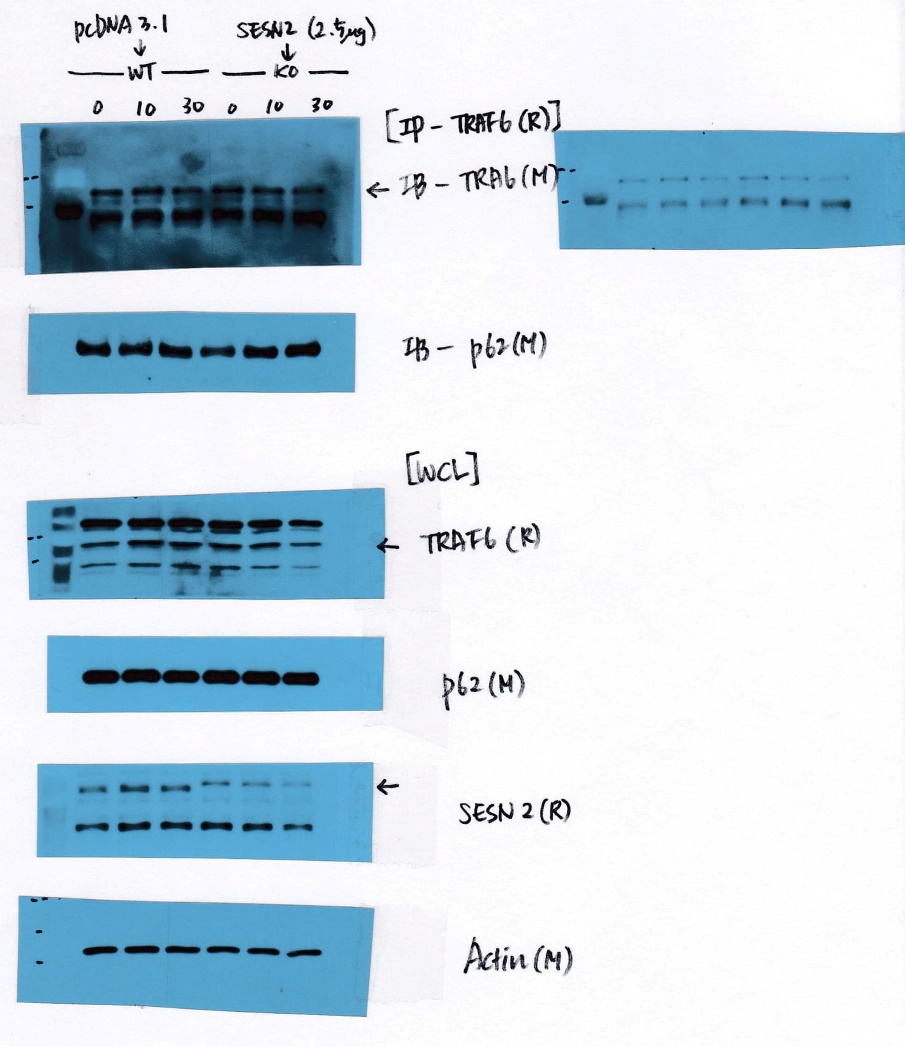

Supplement: Supplementary file 1 [file Table_1.DOCX]
